# A randomized trial of a multimodal lifestyle intervention in cancer survivors

**DOI:** 10.3389/fonc.2025.1682244

**Published:** 2025-10-02

**Authors:** Justin C. Brown, Phillip Nauta, Darryl Whitehead, Benjamin R. Dubin, Ryan Nash, Kate Blumberg, Tamara Green, John Brown, Stephanie L. E. Compton, Gerald P. Miletello

**Affiliations:** ^1^ AdventHealth, Orlando, FL, United States; ^2^ Pennington Biomedical Research Center, Baton, Rouge, LA, United States; ^3^ Franciscan Missionaries of Our Lady Health System, Baton Rouge, LA, United States

**Keywords:** obesity, exercise, cancer, lifestyle modification, weight loss, cardiovascular fitness

## Abstract

**Introduction:**

Cancer survivors are often insufficiently physically active, have overweight or obesity, and suboptimal cardiorespiratory fitness. The Small Steps study evaluated a multimodal intervention to address these modifiable risk factors.

**Methods:**

The study randomized 33 cancer survivors to a 10-week multimodal lifestyle intervention (MLI) of exercise training and nutritional counseling or waitlist control (WLC). The co-primary endpoints included body weight and cardiorespiratory fitness capacity; secondary and exploratory endpoints included cardiometabolic and patient-reported measures. Endpoints were analyzed using analysis of covariance.

**Results:**

Participants had a mean (SD) age of 60.3 (14.0) years, 26 (79%) were White, and 18 (55%) were survivors of breast cancer. At baseline, the mean body weight was 94.9 (18.3) kg, and the submaximal cardiopulmonary fitness was 16.4 (5.0) mL/kg/min. As compared with WLC, MLI reduced body weight [−2.3 kg (95% CI: −3.6, −0.9); P = 0.0013; −2.8% (95% CI: −4.3, −1.3)] and increased cardiopulmonary fitness [2.0 mL/kg/min (95% CI: 0.3, 3.8); P = 0.022]. MLI reduced waist circumference [−2.9 cm (95% CI: −5.5, −0.3); P = 0.029], fat mass [−1.7 kg (95% CI: −2.9, −0.5); P = 0.005], visceral adipose tissue [−168.0 cm^3^ (95% CI: −380.4, −27.7); P = 0.019], and improved self-reported vitality [12.2 points (95% CI: 1.6, 22.8); P = 0.024] and social functioning [14.2 points (95% CI: 1.1, 27.4); P = 0.034]. MLI did not reduce lean mass [−0.2 kg (95% CI: −0.8, 0.4); P = 0.52] or bone mineral density [0.004 g/cm^3^ (95% CI: −0.012, 0.020); P = 0.63]. There were no serious adverse events.

**Discussion:**

The Small Steps program reduced body weight and improved cardiopulmonary fitness in survivors of various types of cancer. This program may contribute to improved health span after cancer.

**Clinical trial registration:**

clinicaltrials.gov, identifier NCT04987359.

## Introduction

There are 18.1 million cancer survivors in the United States, and by the year 2040, this estimate is predicted to increase to 26 million (1). Despite living longer after cancer (2), patients experience myriad symptoms, side effects, and medical complications as a result of cancer and cancer treatment (3). Compared with the general population, cancer survivors are susceptible to cardiovascular disease (4–6), diabetes (7), functional impairment (8), and poor quality of life (9), which collectively erode healthspan (10).

Cancer survivors are often insufficiently physically active (11), have overweight or obesity (12), and suboptimal cardiorespiratory fitness (13). The diagnosis of cancer may offer a teachable moment for health promotion and risk factor reduction (14, 15). Cancer survivors often wish to understand how changing their lifestyle will impact how they feel, function, and survive (16), and oncologists recognize the importance of physical activity, weight management, and diet for their patients (17). However, interventions to address these modifiable risk factors are underutilized in clinical practice because of time constraints, lack of established reimbursement schedules, and limited expertise to offer precise recommendations (18).

The Small Steps clinical study evaluated a 10-week multimodal lifestyle intervention (MLI), compared with a waitlist control (WLC), on co-primary endpoints of cardiorespiratory fitness capacity and body weight in a diverse population of cancer survivors. The multimodal lifestyle program utilized behavioral therapy with structured exercise training and nutritional counseling. This manuscript reports the primary, secondary, and exploratory endpoints of the Small Steps trial that were needed to inform the design and implementation of an evidence-based standard of care clinical program to optimize cancer survivorship.

## Methods

### Study design

The study used a randomized, parallel-group, controlled design conducted at a single site. The study followed good clinical practice and ethical principles in the Declaration of Helsinki. All study activities were approved by an Institutional Review Board. All participants provided written informed consent before completing study activities. The study was registered on ClinicalTrials.gov as NCT04987359.

### Subjects

Eligible participants were adults (aged 18 years and older) with a history of any type of invasive cancer who had completed cancer-directed therapy (e.g., surgery, chemotherapy, and radiotherapy) prior to enrollment (patients receiving ongoing endocrine therapy, targeted therapy, or immunotherapy were eligible). All participants had a body mass index (BMI) ≥30 kg/m^2^ or BMI ≥27 kg/m^2^ with one or more treated or untreated weight-related coexisting conditions. Participants had no contraindications to physical activity, as determined by a physical activity readiness questionnaire (19). Eligible participants engaged in ≤3 exercise bouts per week, on average, over the past 12 weeks, were weight stable, and did not currently use medications or devices for the purpose of weight loss. Patients with a history of metabolic or bariatric surgery were not eligible.

### Random assignment and blinding

Participants were randomly assigned in a 1:1 ratio to WLC or MLI using simple randomization generated with a random number generator. Outcome assessors were blinded to treatment assignment, but participants and intervention staff were not blinded.

### Waitlist control group

Participants assigned to the WLC group were asked to maintain their current exercise and dietary habits for the 10-week study period. Upon providing study endpoint data at week 10, participants assigned to the WLC group were offered a complimentary four-week program, like that of the MLI group.

### Multimodal lifestyle intervention group

Participants assigned to the MLI group received intensive behavioral therapy with structured exercise training and nutritional counseling. The main objective of the structured exercise training was to improve cardiorespiratory fitness. The main objective of nutritional counseling was to improve diet quality and promote a modest calorie deficit to induce weight loss aimed at maximizing reductions in body fat while limiting catabolism of lean mass. Exercise training was three days per week, at moderate intensity (e.g., ≥40 to ≤84% of the heart rate reserve), and progressively titrated to 180 minutes per week, as tolerated. The duration and intensity of each exercise bout were measured with a heart rate monitor. Nutritional counseling included individualized in-person treatment, once per week, for the first four weeks, and alternating weeks thereafter (7 total sessions over 10 weeks). Nutritional counseling consisted of behavior change and motivational interviewing techniques, consistent with the Diabetes Prevention Program guidelines (20).

### Outcome measures

Assessment of anthropometric and body composition measures followed standardized procedures in a fasted state. Participants wore a medical gown and were asked to remove their shoes and jewelry. Height was measured using a wall-mounted stadiometer. Weight was measured in duplicate using a calibrated digital scale; a third measure was obtained if the difference between the first two measures exceeded 0.5 kg. Waist circumference was measured midway between the lower rib margin and the iliac crest, and hip circumference was measured at the widest circumference around the buttocks; measurements were obtained in duplicate, and a third measure was obtained if the difference between the first two measures exceeded 0.5 cm. Body composition was measured with whole-body dual-energy x-ray absorptiometry (DXA; GE). Cardiorespiratory fitness was assessed by a submaximal cardiopulmonary exercise test using a modified Bruce protocol on an electronic motorized treadmill, with expired gases analyzed continuously by a calibrated metabolic measurement system until 80% of the age-predicted maximal heart rate was achieved (Parvo Medics, TrueOne 2400) (21). Systolic and diastolic blood pressure and heart rate were measured in triplicate after five minutes of quiet seated rest. The Short Form 36 (SF-36) evaluated quality of life (22).

### Statistical analysis

The sample size was selected to provide sufficient statistical power to demonstrate treatment differences for the two co-primary endpoints, cardiorespiratory fitness capacity and body weight. The type-I error rate was distributed across the two co-primary outcomes to maintain the overall type-I error rate at 5%. Assuming a standard deviation for change in cardiorespiratory fitness capacity of 3.2 mL/kg/min, 44 participants provided 80% power to detect a 2.8 mL/kg/min difference between randomized groups with the type-I error rate controlled at 4.5% (α=0.045). Assuming a standard deviation for percentage weight loss of 4.8% (23, 24), 44 participants provided 80% power to detect a 5.5% difference between randomized groups with the type-I error rate controlled at 0.5% (α=0.005). Accrual was stopped after enrolling the first 33 participants because the principal investigator (Brown) changed academic institutions. The primary analysis of continuous variable endpoints was conducted with an analysis of covariance model with the baseline value of the dependent variable included as a covariate (25). Model fit was assessed using standard methods. Estimated treatment differences are reported as means and 95% confidence intervals with corresponding P values. Repeated measures correlations were conducted for hypothesis generation using the methods described by Bland and Altman (26).

## Results

### Baseline characteristics

**Figure 1:**
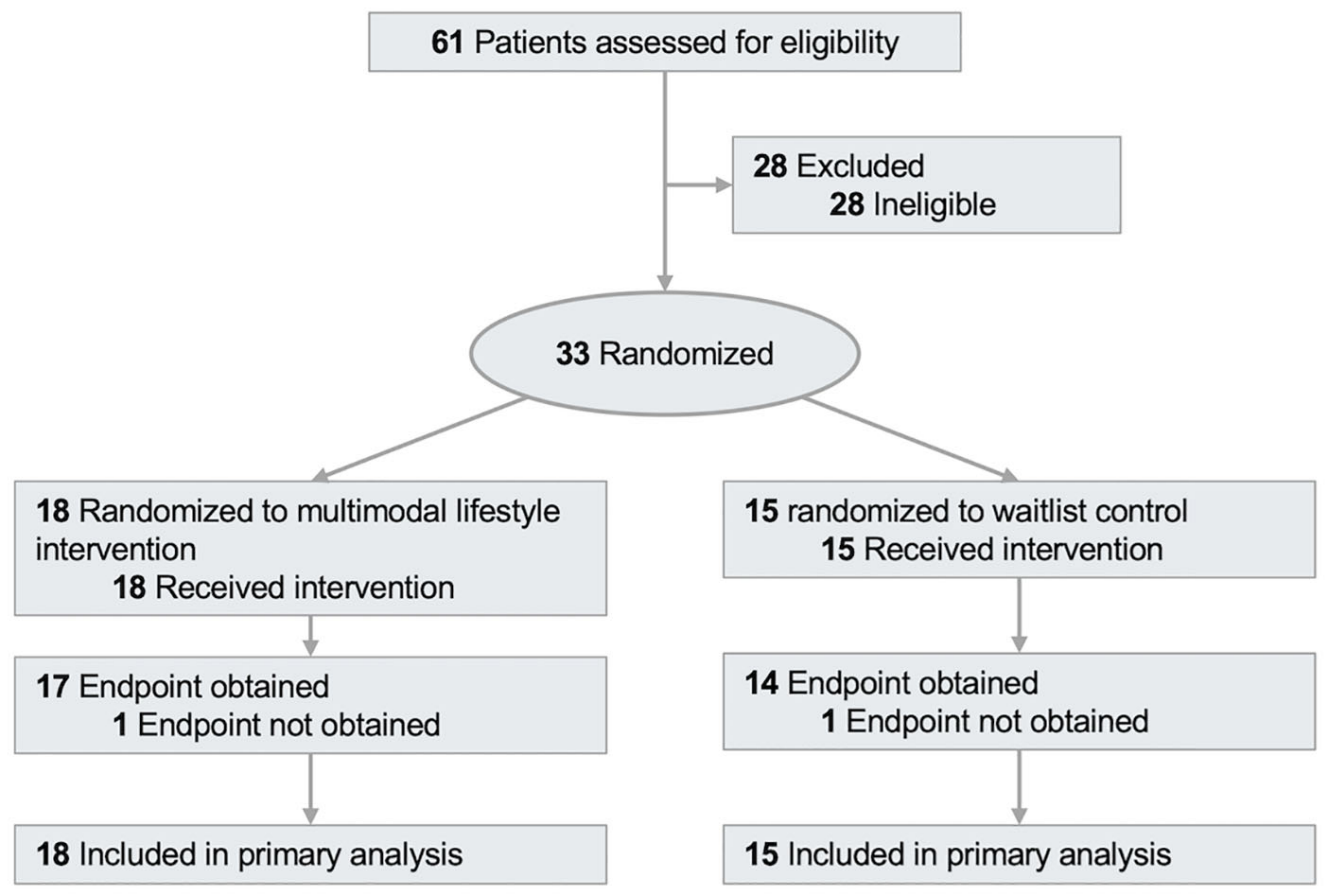
Participant flow through the study.

Between October 2021 and February 2025, 61 patients were screened for eligibility, and 33 were randomized (**Figure 1**). Study participants had a mean (SD) age of 60.3 (14.0) years (**Table 1**), 26 (79%) were of white race, and 18 (55%) were survivors of breast cancer. At baseline, medication was used for hypertension [11 participants (33%)], cholesterol [9 participants (27%)], and diabetes [6 participants (18%)].

**Table 1:** Baseline participant characteristics.

Characteristic	Multimodal Lifestyle Intervention [MLI; *n* = 18]	Waitlist Control [WLC; *n* = 15]
Age, y	60.2±16.5	60.5±10.8
Sex, *n* (%)		
Male	5 (28%)	3 (20%)
Female	13 (72%)	12 (80%)
Race, *n* (%)		
White	16 (89%)	10 (67%)
Black	2 (11%)	5 (33%)
Ethnicity, *n* (%)		
Non-Hispanic	18 (100%)	14 (93%)
Hispanic	0 (0%)	1 (7%)
Cancer site, *n* (%)		
Breast	10 (56%)	8 (53%)
Gynecologic	1 (6%)	2 (13%)
Hematologic	2 (11%)	1 (7%)
Cutaneous	1 (6%)	1 (7%)
Gastrointestinal	2 (11%)	0 (0%)
Genitourinary	1 (6%)	1 (7%)
Endocrine	0 (0%)	1 (7%)
Sarcoma	0 (0%)	1 (7%)
Thoracic	1 (6%)	0 (0%)
Surgery, *n* (%)		
Yes	16 (89%)	14 (93%)
No	2 (11%)	1 (7%)
Chemotherapy, *n* (%)		
Yes	8 (44%)	5 (33%)
No	10 (56%)	10 (67%)
Radiotherapy, *n* (%)		
Yes	7 (39%)	3 (20%)
No	11 (61%)	12 (80%)
Hormonal therapy, *n* (%)		
Yes	4 (22%)	7 (47%)
No	14 (78%)	8 (53%)
Cholesterol medication, *n* (%)		
Yes	6 (33%)	3 (20%)
No	12 (67%)	12 (80%)
Hypertension medication, *n* (%)		
Yes	7 (39%)	4 (27%)
No	11 (61%)	11 (73%)
Diabetes medication, *n* (%)		
Yes	4 (22%)	2 (13%)
No	14 (78%)	13 (87%)

### Multimodal lifestyle intervention adherence

Participants randomized to the MLI group completed 87±16% of the prescribed exercise sessions and 97±1% of the prescribed nutritional counseling sessions.

### Co-primary endpoints

At baseline, the mean bodyweight was 94.9 (18.3) kg, and BMI was 34.0 (5.2) kg/m^2^; 27 participants (67%) had obesity (BMI ≥30 kg/m^2^), of whom 9 (33%) had severe obesity (BMI ≥35 kg/m^2^). At week 10, as compared with WLC, MLI statistically significantly reduced body weight [−2.3 kg (95% CI: −3.6, −0.9); P = 0.0013; −2.8% (95% CI: −4.3, −1.3); −0.8 kg/m^2^ (95% CI: −1.3, −0.3); **Table 2**]. At baseline, the mean submaximal cardiopulmonary fitness was 16.4 (5.0) mL/kg/min. At week 10, as compared with WLC, MLI statistically significantly increased cardiopulmonary fitness [2.0 mL/kg/min (95% CI: 0.3, 3.8); P = 0.022].

**Table 2:** Coprimary, secondary, and exploratory cardiometabolic endpoints.

Endpoint	Randomized group	Baseline (mean±SD)	Δ Baseline to week 10 (mean±SE)	Δ Between group (mean, 95% CI)	P
Coprimary endpoints					
Bodyweight, kg	WLC	96.4±16.0	−0.3±0.5	—	—
	MLI	93.7±20.5	−2.5±0.5	−2.3 (−3.6, −0.9)	0.0013
Fitness, mL/kg/min	WLC	15.5±4.9	−0.7±0.6	—	—
	MLI	17.2±5.2	1.3±0.6	2.0 (0.3, 3.8)	0.022
Secondary endpoints					
Waist circumference, cm	WLC	110.0±11.8	0.1±1.0	—	—
	MLI	108.9±14.3	−2.8±0.9	−2.9 (−5.5, −0.3)	0.029
Fat mass, kg	WLC	45.5±9.4	−0.2±0.4	—	—
	MLI	43.0±13.0	−1.9±0.4	−1.7 (−2.9, −0.5)	0.005
Systolic blood pressure, mmHg	WLC	120.9±11.1	−1.3±3.1	—	—
	MLI	119.1±11.4	0.3±3.0	1.6 (−6.8, 10.0)	0.71
Diastolic blood pressure, mmHg	WLC	70.3±7.5	0.2±2.2	—	—
	MLI	69.0±7.4	−0.4±2.1	−0.6 (−6.5, 5.2)	0.84
Exploratory endpoints					
Bodyweight, %	WLC	96.4±16.0	−0.1±0.6	—	—
	MLI	93.7±20.5	−3.0±0.5	−2.8 (−4.3, −1.3)	0.001
Body mass index, kg/m^2^	WLC	34.5±4.2	−0.1±0.2	—	—
	MLI	33.5±6.1	−0.9±0.2	−0.8 (−1.3, −0.3)	0.003
Hip circumference, cm	WLC	120.8±11.2	−0.4±0.8	—	—
	MLI	116.9±12.2	−2.2±0.8	−1.8 (−4.1, 0.4)	0.104
Waist-to-hip circumference, ratio	WLC	0.91±0.09	0.00±0.01	—	—
	MLI	0.93±0.09	−0.01±0.01	−0.01 (−0.03, 0.01)	0.30
Body fat percentage, %	WLC	48.8±5.2	−0.1±0.3	—	—
	MLI	47.4±7.5	−1.2±0.3	−1.1 (−1.9, −0.3)	0.006
Visceral adipose volume, cm^3^	WLC	2200±1408	81.6±53.0	—	—
	MLI	2193±1414	−86.4±48.1	−168.0 (−308.4, −27.7)	0.019
Lean mass, kg	WLC	47.6±9.4	0.1±0.2	—	—
	MLI	46.9±10.8	−0.1±0.2	−0.2 (−0.8, 0.4)	0.52
Bone mineral density, g/cm^3^	WLC	1.14±0.10	−0.002±0.006	—	—
	MLI	1.19±0.15	0.002±0.005	0.004 (−0.012, 0.020)	0.63
Resting heart rate, bpm	WLC	66.8±8.2	2.8±2.5	—	—
	MLI	67.1±9.6	−0.3±2.3	−3.1 (−9.7, 3.6)	0.37

Secondary and exploratory endpoints are not adjusted for multiple hypothesis testing.

### Secondary endpoints

At week 10, as compared with WLC, MLI statistically significantly reduced waist circumference [−2.9 cm (95% CI: −5.5, −0.3); P = 0.029] and total-body fat mass [−1.7 kg (95% CI: −2.9, −0.5); P = 0.005], but did not change systolic blood pressure [1.6 mmHg (95% CI: −6.8, 10.0); P = 0.71] or diastolic blood pressure [−0.6 mmHg (95% CI: −6.5, 5.2); P = 0.84]. At week 10, as compared with WLC, MLI did not change patient-reported overall physical health [2.1 points (95% CI: −4.9, 9.2); P = 0.55; **Table 3**] or mental health [8.4 points (95% CI: −1.3, 18.2); P = 0.089]. One participant, randomized to MLI, reported stopping taking medication for hypertension (P = 0.81); no other changes in medication use were reported in either group.

**Table 3:** Secondary and exploratory patient-reported endpoints.

Endpoint	Randomized group	Baseline (mean±SD)	Δ Baseline to week 10 (mean±SE)	Δ Between group (mean, 95% CI)	P
Secondary endpoints					
Physical health summary	WLC	79.2±13.3	−1.0±2.6	—	—
	MLI	75.9±14.6	1.2±2.5	2.1 (−4.9, 9.2)	0.55
Mental health summary	WLC	82.6±11.3	−5.2±3.6	—	—
	MLI	79.2±10.3	3.3±3.4	8.4 (−1.3, 18.2)	0.089
Exploratory endpoints					
Physical functioning	WLC	81.3±17.1	3.1±2.4	—	—
	MLI	80.8±15.0	−0.3±2.2	−3.4 (−9.8, 3.0)	0.30
Role—physical	WLC	88.3±26.5	−4.6±8.0	—	—
	MLI	82.4±29.8	−0.2±7.6	4.5 (−17.2, 26.1)	0.69
Bodily pain	WLC	83.8±15.1	−4.7±3.9	—	—
	MLI	79.6±20.4	1.0±3.6	5.7 (−4.8, 16.2)	0.29
General health	WLC	63.3±15.1	0.4±3.0	—	—
	MLI	60.6±17.8	5.3±2.8	4.9 (−3.2, 12.9)	0.23
Vitality	WLC	60.7±19.5	−3.7±3.9	—	—
	MLI	54.4±20.1	8.5±3.7	12.2 (1.6, 22.8)	0.024
Social functioning	WLC	95.8±7.7	−9.3±4.9	—	—
	MLI	87.5±15.5	5.0±4.6	14.2 (1.1, 27.4)	0.034
Role—emotional	WLC	91.1±23.4	−7.5±8.5	—	—
	MLI	90.7±22.3	0.2±8.1	7.7 (−15.3, 30.7)	0.51
Mental health	WLC	82.7±10.4	−0.3±2.6	—	—
	MLI	84.0±8.6	0.1±2.5	0.4 (−6.7, 7.4)	0.92

Secondary and exploratory endpoints are not adjusted for multiple hypothesis testing.

### Exploratory endpoints

At week 10, as compared with WLC, MLI statistically significantly reduced body fat percentage [−1.1% (95% CI: −1.9, −0.3); P = 0.006], visceral adipose tissue [−168.0 cm^3^ (95% CI: −380.4, −27.7); P = 0.019], and statistically significantly improved self-reported vitality [12.2 points (95% CI: 1.6, 22.8); P = 0.024] and social functioning [14.2 (1.1, 27.4); P = 0.034].

At week 10, as compared with WLC, MLI did not change hip circumference [−1.8 cm (95% CI: −4.1, 0.4); P = 0.104], the ratio of the waist-to-hip circumferences [−0.01 (95% CI: −0.03, 0.01); P = 0.30], lean mass [−0.2 kg (95% CI: −0.8, 0.4); P = 0.52], bone mineral density [0.004 g/cm^3^ (95% CI: −0.012, 0.020); P = 0.63], or resting heart rate [−3.1 beats/minute (95% CI: −9.7, 3.6); P = 0.37]. At week 10, as compared with WLC, MLI did not change patient-reported physical functioning subscale score [−3.4 points (95% CI: −9.8, 3.0); P = 0.30], role—physical subscale score [4.5 points (95% CI: −17.2, 26.1); P = 0.69], bodily pain subscale score [5.7 points (95% CI: −4.8, 16.2); P = 0.23], general health subscale score [4.9 points (95% CI: −3.2, 12.9); P = 0.23], role—emotional subscale score [7.7 points (95% CI: −15.3, 30.7); P = 0.51], or mental health subscale score [0.4 points (95% CI: −6.7, 7.4); P = 0.92].

### Exploratory correlational analyses

Longitudinal changes in body weight correlated with changes in submaximal cardiopulmonary fitness [ρ=−0.41 (95% CI: −0.70, −0.01); P = 0.035]. Longitudinal correlation analyses of the co-primary endpoints with secondary and exploratory endpoints are provided for hypothesis-generating purposes (**Supplementary Table 1**).

### Adverse events

No participants reported any serious or unexpected adverse events.

## Discussion

This trial evaluated the effects of 10 weeks of structured exercise training and nutritional counseling in cancer survivors who were insufficiently physically active and had overweight or obesity at baseline. Compared with the WLC group, participants randomized to the MLI group experienced statistically significant improvements in the co-primary outcomes: reduction in body weight and increase in cardiorespiratory fitness. Additionally, the MLI group experienced favorable changes in body composition, improvements in select quality of life metrics, without negatively affecting lean tissue or bone mineral density. The findings from this randomized trial contribute preliminary data to the growing body of evidence supporting lifestyle modification as an important component of comprehensive cancer survivorship care (27).

The co-primary endpoints of this trial were selected because cancer survivors have higher rates of overweight and obesity and lower cardiopulmonary fitness than their age-matched peers without cancer (12, 13). Participants randomized to the MLI group lost an average of 2.3 kg (2.8% of baseline body weight) at week 10, compared with the WLC group. Although modest in magnitude, this amount of weight loss is consistent with early clinical benefits in metabolic health (28). The average adult gains approximately ~0.5 kg of body weight annually (29); therefore, this modest weight loss may promote weight stability longitudinally. In observational studies, obesity is associated with an increased risk of cancer recurrence (30), and weight gain, compared with weight stability, is associated with limitations in activities of daily living and an increased risk of cancer-specific mortality (31, 32). Participants randomized to the MLI group gained an average of 2 mL/kg/min in cardiopulmonary fitness at week 10, compared with the WLC group. In observational studies, each 1 mL/kg/min increase in cardiopulmonary fitness is associated with a ~7% relative reduction in the risk of death in cancer survivors (33).

With respect to the secondary and exploratory physiological study endpoints, participants randomized to the MLI group experienced statistically significant reductions in waist circumference, total body fat mass, and visceral adipose tissue volume estimated using DXA, highlighting the impact on central adiposity. These physiological changes may have favorable implications for quality of life, cancer prognosis, and competing causes of morbidity and mortality in cancer survivors, such as cardiovascular disease and diabetes (34). Importantly, the MLI group preserved lean mass and bone mineral density, addressing a major concern in lifestyle interventions that weight loss can accelerate sarcopenia and osteoporosis, particularly in aging cancer survivors. The study did not observe changes in resting heart rate or blood pressure, possibly because baseline values were within normal limits, which impeded our ability to detect treatment effects.

With respect to the secondary and exploratory quality of life endpoints, participants randomized to the MLI group experienced statistically significant improvements in vitality and social functioning, which are often impaired in cancer survivors (35). Exploratory correlational analyses revealed an association between weight loss and patient-reported general health, such that as weight was reduced, general health improved, and significant associations between cardiopulmonary fitness and vitality, such that as fitness increased, vitality improved. These findings suggest that body weight and cardiopulmonary fitness may serve as surrogates or mediators of psychosocial health benefits in this population. The results of these secondary and exploratory physiological and quality of life endpoints should be interpreted cautiously, as we did not control the type I error rate, which may contribute to false positive findings and unstable effect size estimates, and the smaller than intended sample size may inflate the type II error rate, which may contribute to false negative findings.

The results of the Small Steps study can be compared with those of a similar trial, the Healthy Living After Cancer (HLAC), conducted at Dana-Farber Cancer Institute (24). HLAC was a 15-week trial that randomized 60 cancer survivors to a group-based weight loss intervention that included calorie restriction and physical activity or a waitlist control group. At week 15, the intervention produced 5.6% weight loss, reduced fat mass, and increased objectively measured physical functioning (24). Key distinctions between HLAC and Small Steps are group-based versus participant-based behavioral counseling, and supervised exercise versus home-based physical activity. These trials contribute randomized real-world evidence regarding the myriad general health benefits and safety and feasibility of lifestyle modification for cancer survivors (36, 37). The framework used in the Small Steps trial could be adapted for community or academic cancer centers through models such as shared medical visits, telehealth coaching, or partnerships with local fitness and nutrition programs. By targeting weight reduction and cardiorespiratory fitness, lifestyle interventions may mitigate the long-term sequelae of cancer treatment and improve survivorship outcomes.

There are several important limitations to this study. The sample size was modest (n=33), and accrual was terminated prematurely due to institutional changes. This limits statistical power and increases the risk of a type II error (false negative) for secondary and exploratory endpoints. The secondary and exploratory endpoints were not adjusted for multiple comparisons, increasing the likelihood of false-positive findings. Although the primary endpoints were appropriately powered and adjusted for type I error, caution is warranted when interpreting other statistically significant outcomes. Although racially diverse (24% Black participants), the study population was predominantly composed of female participants with breast cancer. This reflects real-world survivorship demographics (38), but limits generalizability to male survivors and those with less common cancers. The study population was recruited from a single site in the southern part of the United States, and therefore, the generalizability of these results to other regions within the United States or other countries is not known. The 10-week intervention duration may be too short to observe maximal or sustained benefits, as many physiologic and patient-reported outcomes may require longer durations to achieve the maximal magnitude of benefit. Future studies will be needed to assess the maintenance of behavior change and resultant health benefits.

There are several key strengths to this study. First, its randomized design with blinded outcome assessors minimizes bias and supports causal inference. Second, the use of objective measures, such as DXA for body composition and treadmill-based submaximal testing for fitness, lends rigor to the physiological outcomes. Third, adherence rates were high—87% for supervised exercise sessions and 97% for nutritional counseling, highlighting the acceptability and feasibility of the intervention in a clinical survivorship setting. Additionally, the MLI was delivered by trained professionals using behavioral therapy principles grounded in evidence-based frameworks (e.g., Diabetes Prevention Program), increasing the potential for scalability and standardization. Importantly, no serious or unexpected adverse events occurred, confirming the safety of MLI even in a heterogeneous survivor population.

In conclusion, the Small Steps randomized trial demonstrated that a 10-week multimodal lifestyle intervention—combining supervised exercise and individualized nutritional counseling—produced improvements in body weight, cardiorespiratory fitness, and markers of body composition in cancer survivors who were insufficiently physically active and had overweight or obesity at baseline. The intervention was safe, feasible, and associated with high adherence and favorable changes in patient-reported vitality and social functioning. These results support the feasibility of conducting larger definitive trials that embed lifestyle interventions into cancer survivorship care. While further research is needed to evaluate long-term outcomes, cost-effectiveness, and scalability, the Small Steps program represents a promising, evidence-based model that may help cancer survivors not only survive but thrive.

## Data Availability

The raw data supporting the conclusions of this article will be made available by the authors, without undue reservation.
