# Supplementary material for: A randomized trial of a multimodal lifestyle intervention in cancer survivors
Source: Front Oncol. 2025 Oct 2;15:1682244. doi: 10.3389/fonc.2025.1682244 (PMC12527883; doi:10.3389/fonc.2025.1682244)
Supplement: Supplementary file 1 [file Table1.docx]

**A Randomized Trial of a Multimodal Lifestyle Intervention in Cancer Survivors**

Justin C. Brown^1^; Phillip Nauta^2^; Darryl Whitehead^2^; Benjamin R Dubin^2^; Ryan Nash^2^; Kate Blumberg^2^; Tamara Green^2^; John Brown^2^; Stephanie L.E. Compton^1^; Gerald P. Miletello^3^

^1^AdventHealth, Orlando, FL, 32804; ^2^Pennington Biomedical Research Center, Baton Rouge, LA, 70808; ^3^Franciscan Missionaries of Our Lady Health System, Baton Rouge, LA 70809

**Corresponding Author:**

Justin C. Brown, Ph.D.

AdventHealth

301 E Princeton St

Orlando, FL 32804, USA

Email: Justin.Brown4@AdventHealth.com

**Running Head:** Lifestyle in Cancer Survivorship

**Disclosures:** Dr. Brown reports receiving grants paid to his employer from the National Institutes of Health and the American Institute for Cancer Research. Dr. Brown reports receiving speaking fees from Nestlé Health Science within the past 24 months. Other authors had no disclosures.

**Supplementary Table 1**. Longitudinal (repeated measures) correlation analyses of coprimary endpoints with secondary and exploratory endpoints in the randomized sample (*n* = 33)

| **Variable** | **Bodyweight** | **Fitness** |
| --- | --- | --- |
| Waist circumference | ρ=0.56; 95% CI: 0.23, 0.77*** | ρ=−0.39; 95% CI: −0.69, 0.03 |
| Fat mass | ρ=0.89; 95% CI: 0.78, 0.95*** | ρ=−0.43; 95% CI: −0.72, −0.02* |
| Systolic blood pressure | ρ=0.16; 95% CI: 0.23, 0.50 | ρ=−0.22; 95% CI: −0.58, 0.21 |
| Diastolic blood pressure | ρ=0.39; 95% CI: 0.01, 0.67* | ρ=−0.18; 95% CI: −0.55, 0.24 |
| Body mass index | ρ=0.99; 95% CI: 0.98, 0.99*** | ρ=−0.40; 95% CI: −0.69, −0.01* |
| Hip circumference | ρ=0.61; 95% CI: 0.31, 0.80*** | ρ=−0.47; 95% CI: −0.74, −0.07* |
| Waist-to-hip circumference | ρ=0.16; 95% CI: −0.22, 0.50 | ρ=−0.05; 95% CI: −0.45, 0.37 |
| Body fat percentage | ρ=0.77; 95% CI: 0.55, 0.88*** | ρ=−0.42; 95% CI: −0.71, −0.01* |
| Visceral adipose volume | ρ=0.45; 95% CI: 0.10, 0.71** | ρ=−0.17; 95% CI: −0.54, 0. 25 |
| Lean mass | ρ=0.16; 95% CI: −0.22, 0.50 | ρ=0.13; 95% CI: −0.30, 0.51 |
| Bone mineral density | ρ=−0.06; 95% CI: −0.43, 0.31 | ρ=0.07; 95% CI: −0.35, 0.47 |
| Resting heart rate | ρ=0.01; 95% CI: −0.36, 0.38 | ρ=0.03; 95% CI: −0.39, 0.44 |
| Physical health summary | ρ=−0.10; 95% CI: −0.46, 0.28 | ρ=0.10; 95% CI: −0.33, 0.49 |
| Mental Health Summary | ρ=−0.08; 95% CI: −0.44, 0.29 | ρ=0.29; 95% CI: −0.13, 0.63 |
| Physical functioning | ρ=0.07; 95% CI: −0.31, 0.43 | ρ=0.09; 95% CI: −0.34, 0.48 |
| Role⎯physical | ρ=0.01; 95% CI: −0.36, 0.38 | ρ=0.05; 95% CI: −0.37, 0.45 |
| Bodily pain | ρ=0.10; 95% CI: −0.29, 0.46 | ρ=−0.16; 95% CI: −0.53, 0.27 |
| General health | ρ=−0.37; 95% CI: −0.65, −0.01* | ρ=0.27; 95% CI: −0.16, 0.61 |
| Vitality | ρ=−0.36; 95% CI: −0.65, 0.01 | ρ=0.60; 95% CI: 0.25, 0.81*** |
| Social functioning | ρ=−0.03; 95% CI: −0.40, 0.35 | ρ=0.29; 95% CI: −0.13, 0.63 |
| Role⎯emotional | ρ=0.03; 95% CI: −0.35, 0.40 | ρ=−0.02; 95% CI: −0.43, 0.39 |
| Mental health | ρ=0.06; 95% CI: −0.32, 0.43 | ρ=0.24; 95% CI: −0.19, 0.59 |

*P<0.05; **P<0.01; ***P<0.001
